# Selected Subjects on the Surgery of Infancy and Childhood. Lettsomian Lectures for 1890

**Published:** 1890-12

**Authors:** 


*Selected Subjects on the Surgery of Infancy and Childhood. Lettsomian Lectures for* 1890. By EDMUND OWEN, F.R.C.S. London: BAILLIÈRE & Co. 1890.

There is some picturesque writing and a good deal of sound surgery in the small volume before us. Tropes and figures abound. Thus (p. 5), "setting up inflammation in the surrounding structures, and rendering the skin adherent and undermined, she (Nature) seeks relief from tension by carrying a lighted torch, as it were, among highly inflammable tissues." This process of "rendering the skin undermined" is then compared to what ought to have been in parallel language called "rendering the corn ignited" of the Philistines by Samson vivi-cremating the foxes.

"Keen with the scalpel" (p. 11) may mean more than appears, but it seems rather vulgar. "Picturesque anatomical neighbourhood" (p. 15); "the pneumogastric nerve evinced its resentment by setting up an alarming attack of dyspnœa" (p. 17); "they, and the tissues with which they are continuous, are always ready to pick a quarrel, as it were, with a surgical instrument" (p. 24), are a few of many telling pieces of rhetoric.

The author in operating for hare-lip sometimes leaves the lower lip too prominent. To diminish this prominence, he occasionally removes a "wedge-shape piece from the prominent lower lip." He desires it to be known that this is an original proceeding, and this is the neat way he urges his claim: "I will not presume to say that the idea is a novel one, or I may be favoured with a dozen strongly-worded notes to the effect that the plan has been resorted to in numberless bad cases since it was first recommended by some native Indian surgeon whose name it is difficult to spell, or by some

Russian operator whose name it is difficult to pronounce." "What I know not, is not knowledge," has recently been assumed by London surgeons other than the author. In this case, however, we honestly believe the author is safe: no surgeon is likely to dispute with him the honour of introducing an operation which, after doing away with too much of the upper lip, seeks to balance the fault by removing a piece of the lower. It is well, however, to know that a Lettsomian lecturer may indulge in the "playful-sarcastic."

The author is clearly a capable and original surgeon. Whether these lectures are worthy of the audience and the Founder, we do not presume to decide. We confess that we like to see lectures made interesting by means other than our author has adopted.

